# A Mixed Methods Pilot Study of an Equity‐Explicit Student‐Teacher Relationship Intervention for the Ninth‐Grade Transition

**DOI:** 10.1111/josh.12968

**Published:** 2020-11-12

**Authors:** Larissa M. Gaias, Clayton R. Cook, Lillian Nguyen, Stephanie K. Brewer, Eric C. Brown, Sharon Kiche, Jiajing Shi, Jodie Buntain‐Ricklefs, Mylien T. Duong

**Affiliations:** ^1^ Assistant Professor, (larissa_gaias@uml.edu), Department of Psychology University of Massachusetts Lowell, 850 Broadway St Lowell, MA 01854; ^2^ Professor, (crcook@umn.edu), College of Education and Human Development University of Minnesota, 56 East River Road, Minneapolis, MN 55455; ^3^ Project Coordinator, (lilliann@utexas.edu), School Mental Health Assessment, Research, and Training Center University of Washington, 6200 NE 74^th^ St, Seattle, WA 98115; ^4^ Postdoctoral Fellow, (sbrewer1@uw.edu), School Mental Health Assessment, Research, and Training Center University of Washington, 6200 NE 74^th^ St, Seattle, WA 98115; ^5^ Associate Professor, (ecb41@miami.edu), Miller School of Medicine University of Miami, 1600 NW 10^th^ Ave, Miami, FL 33136; ^6^ Project Coordinator, (kichews@uw.edu), School Mental Health Assessment, Research, and Training Center University of Washington, 6200 NE 74^th^ St, Seattle, WA 98115; ^7^ Research Study Assistant, (sshi@cfchildren.org), Committee for Children, 2815 2^nd^ Ave, Seattle, WA 98121; ^8^ Research Lead/Managing Director, (jjbr@uw.edu), School Mental Health Assessment, Research, and Training Center University of Washington, 6200 NE 74^th^ St, Seattle, WA 98115; ^9^ Senior Research Scientist, (mduong@cfchildren.org) Committee for Children, 2815 2^nd^ Ave, Seattle, WA 98121

**Keywords:** student‐teacher relationships, education equity, implicit bias, transition to high school transition, teacher professional development, school program evaluation

## Abstract

**BACKGROUND:**

Student‐teacher relationships are associated with the social and emotional climate of a school, a key domain of the Whole School, Whole Community, Whole Child Model. Few interventions target student‐teacher relationships during the critical transition to high school, or incorporate strategies for enhancing equitable relationships. We conducted a mixed‐methods feasibility study of a student‐teacher relationship intervention, called Equity‐Explicit Establish‐Maintain‐Restore (E‐EMR).

**METHODS:**

We tested whether students (N = 133) whose teachers received E‐EMR training demonstrated improved relationship quality, school belonging, motivation, behavior, and academic outcomes from pre‐ to post‐test, and whether these differences were moderated by race. We also examined how teachers (N = 16) integrated a focus on equity into their implementation of the intervention.

**RESULTS:**

Relative to white students, students of the color showed greater improvement on belongingness, behavior, motivation, and GPA. Teachers described how they incorporated a focus on race/ethnicity, culture, and bias into E‐EMR practices, and situated their relationships with students within the contexts of their own identity, the classroom/school context, and broader systems of power and privilege.

**CONCLUSIONS:**

We provide preliminary evidence for E‐EMR to change teacher practice and reduce educational disparities for students of color. We discuss implications for other school‐based interventions to integrate an equity‐explicit focus into program content and evaluation.

The Whole School, Whole Community, Whole Child (WSCC) framework has gained increasing attention among researchers and practitioners due to its focus on staff and student health and wellbeing as a necessary condition for promoting academic engagement and school success.^1^ The framework emphasizes 10 interrelated domains that aim to promote 5 proximal indicators of wellbeing outlined by the ASCD's Whole Child Initiative,^2^ including being healthy and feeling supported, challenged, engaged, and safe. In particular, the domain of *social and emotional climate* has strong links to other domains of school health and several of the indicators of staff and student health and wellbeing. A supportive *social and emotional climate* can facilitate students' perceptions of support and safety within their schools and improve mental, physical, and behavioral health outcomes;^3^, ^4^, ^5^ teachers also benefit from a promotive social and emotional climate, through reductions in stress, depressive symptoms, and burnout and increased feelings of support.^6^, ^7^, ^8^A core component of social and emotional climate is students' sense of belonging and connection to school,^9^ which is driven in part by their relationships with their teachers.^10^, ^11^ Inequities exist in this domain, however, with students of color often reporting poorer relationships with teachers and a weaker sense of belonging and connection to school than white students.^12^ This has led some to conclude that opportunity and achievement gaps for students from historically marginalized backgrounds are in part due to relationship gaps.^13^

Frameworks like WSCC offer helpful guidance for schools when planning how to allocate time, energy, and resources to achieve desired outcomes, including increasing school engagement and sense of safety/support that lead to improved academic achievement.^14^, ^15^ For example, when considering the WSCC framework and the critical domain of social and emotional climate, it is important to select programs that introduce practices that improve how students experience school. As discussed above, a core element of social and emotional climate is positive teacher‐student relationships, which promote students' sense of belonging and connection to school.^9^, ^10^, ^11^ The purpose of this study was to conduct a preliminary mixed‐methods evaluation of an equity‐explicit student‐teacher relationship intervention delivered by ninth‐grade teachers for students transitioning into high school. Specifically, we quantitatively examine the intervention's potential to reduce disparities in student outcomes and qualitatively examine teachers' perceptions of the program for enhancing equitable relationship‐building practices. Findings from this study potentially provide additional evidence supporting the WSCC framework in high school settings.

## Importance of Student‐Teacher Relationships

Positive student‐teacher relationships predict short‐^16^ and long‐term academic success,^17^ often mediated by school belonging, student engagement, and improved behavior.^18^, ^19^, ^20^ Student‐teacher relationships may be particularly important in improving a school's social and emotional climate and promoting student outcomes during difficult school transitions.^21^ During the transition to high school, students need to adapt to more challenging and bureaucratic academic and social environments.^22^, ^23^ A student‐teacher relationship is a critical protective factor that can facilitate successful navigation of this challenging transition.^18^, ^24^ High schools whose teachers are highly supportive of students cut the probability of dropping out nearly in half.^24^ However, students spend less time with their teachers beginning in ninth grade and often report feeling poorly supported by teachers and principals,^25^, ^26^ suggesting a critical point for intervention.

It is imperative to integrate an equity perspective when examining different domains of the WSCC, as some students may face disparities with a particular domain, like the social and emotional climate. For example, the relational climate during the transition to high school may be particularly challenging for students of color, who often face greater academic and/or social‐emotional risk factors than white students.^27^, ^28^, ^29^ Although the relation between positive student‐teacher relationships and student outcomes is strongest for students of color,^30^ teachers are least likely to have positive relationships with these students.^31^, ^32^ Cultural or racial/ethnic mismatch between students and their teachers or the school environment can contribute to lower feelings of engagement and belongingness.^33^, ^34^ Teachers may hold implicit biases or explicit stereotypical beliefs regarding students' strengths and weaknesses, which can influence their expectations for students and negatively affect relationships.^35^, ^36^ In addition, systemic discrimination and racism impact school practices and policies, which affect the context where individual relationships develop.^37^ Thus, in addition to targeting the ninth‐grade transition, addressing racial inequities in relationship quality is another critical issue in the need for intervention.

## Interventions Focused on Student‐Teacher Relationships

Consistent with the WSCC framework, interventions that target social and emotional climate indicators are needed at critical transition points that focus on cultivating experiences that buffer against risk and set students on positive trajectories in school. Timely, well‐implemented interventions can facilitate psychological and developmental processes that lead to successful adaptation in the face of change, and trigger a series of reciprocally reinforcing interactions between youth and the school social system.^38^ Students who receive positive attention demonstrate better behavioral and academic outcomes over time, which in turn enhances teachers' perceptions of their capabilities.^39^, ^40^, ^41^

Despite the need to focus on student‐teacher relationships at the high school transition, the vast majority of existing student‐teacher relationship interventions focus on younger students. A notable exception, My Teaching Partner‐Secondary,^42^ has demonstrated promise for improving student outcomes^43^, ^44^ and enhancing equity in the high school setting^45^, ^46^; however, this program involves resource‐intensive coaching and engages in a comprehensive approach to improve instructional interactions beyond an inherent focus on relationships. Establish‐Maintain‐Restore (EMR) is an evidence‐based student‐teacher relationship intervention that is designed to be brief and feasible for implementation in the context of real‐world high schools. Teachers receive training to engage in collaborative processes promoting positive relationships in 3 phases: Establish, Maintain, and Restore (Table 1). These phases serve as a heuristic that guides teachers' decision‐making and practices, with the goal to move all students to the maintain phase of the relationship. The phases are not necessarily linear as a relationship can change depending on the time spent and dynamic interactions between 2 people. Following a training focused on the EMR heuristic and strategies, teachers participate in professional learning communities (PLCs) at their school using a protocol, without a researcher present to facilitate open, honest collaboration, and reflection. During these PLCs, teachers assess their relational status with each student in one of their classes and then develop an action plan for engaging in certain practices with particular students over the next month to move those students into a more favorable relationship phase (ie, maintain). Teachers are encouraged to focus on the students who fall in the Establish and Restore phases.

**Table 1:** Names and Descriptions of E‐EMR Strategies

Practice Name	Practice At‐A‐Glance
Establish	
Banking time	Find individual time to spend with a student to engage in relational conversation
Gather, review, acknowledge	Learn information about students Review that information to combat forgetfulness Find natural opportunities to acknowledge or reference that information
Positive greetings	Use the student's name Welcome student and show that you value their presence
Positive farewells	Offer words of encouragement Say thank you for participating Wish students a good rest of the day
Wise feedback	Explicitly communicate high expectations and reason for feedback Express care for student learning Assure student they are capable of meeting expectations Allow student to advocate for help or provide feedback
2 by 10	Spend 2 minutes/day for 10 days connecting with a student
Objective observations^1^	For students you are struggling with, conduct specific observations Focus on objectively describing the student's behavior, putting aside your interpretations or judgments
Maintain	
5:1 Ratio	Maintain a 5 to 1 positive to negative interaction with each student, including: Effective use of praise Relationship check‐ins Being mindful in the moment^1^
Restore	
Letting go	Fresh start after a negative interaction
Taking ownership	Acknowledge your own mistake/missed opportunity
Empathy statement^1^	Show effort to understand the student's perspective
Statement of care	Separate the deed from the doer
Collaborative problem‐solving	Working together to find win‐win solutions

Note. ^1^indicates strategies for reducing bias and enhancing cultural responsiveness from COR training.

Establish‐Maintain‐Restore was previously tested in small cluster‐randomized trials with elementary^47^ and middle^48^ school teachers. Both trials found improved relationship quality, increased academically engaged time, and decreased disruptive behaviors for students whose teachers received EMR training and participated in PLCs. Results were equivalent for white students and students of color. Thus, although EMR did not exacerbate disparities for students of color, the program did not narrow such disparities either. As the program was adapted for the high school context,^49^ (also Brewer et al, unpublished data), the developers also incorporated an explicit focus on enhancing racial equity by integrating relational strategies for reducing bias and enhancing cultural responsiveness. Before attending the 6‐hour EMR training, teachers in the current study completed a 90‐minute online implicit bias training.^50^ The training described practical strategies for bringing implicit biases into conscious awareness to improve equitable relationship building, which was incorporated into the EMR strategies (Table 1). Also, during the PLCs, teachers reflected on whether students of particular racial/ethnic groups or genders were disproportionately represented across the 3 EMR phases. If so, teachers were encouraged to integrate equity‐explicit strategies into their monthly action plans.

## Current Study

The current study is a preliminary mixed‐methods evaluation of the adapted Equity‐Explicit EMR (E‐EMR) approach designed to support teachers to intentionally cultivate relationships with ninth‐grade students. This evaluation focuses on understanding the potential for E‐EMR to enhance equity in student outcomes and teacher practices. We examine multiple domains of student well‐being aligned with the 5 ASCD WSCC indicators, including feeling engaged, supported, challenged, and safe within their classroom and school environment. Specifically, we were interested in answering these research questions:

- Did students' student‐teacher relationship quality, school belonging, motivation, behavior, and academic outcomes improve from pre‐ to post‐test? Did pre‐ to post‐test changes differ for white students and students of color?
- How did teachers integrate a focus on equity into their use of the E‐EMR strategies? Did teachers perceive E‐EMR to be appropriate and effective for reducing bias and enhancing cultural responsiveness in relationship building?

We engaged in a mixed‐methods component expansion design.^51^ In component designs, the quantitative and qualitative methods are conducted and analyzed independently, and the combining of the 2 data sources occurs during interpretation.^51^ An expansion design emphasizes different methods for distinct components of inquiry.^51^ In this study, we used quantitative methods to understand the potential for E‐EMR to enhance equity in student outcomes and qualitative methods to understand teachers' perspectives regarding E‐EMR implementation.

## METHODS

### Setting and Participants

Participants were recruited from a public high school in the Pacific Northwest. Teachers were eligible to participate if they taught at least 50% ninth‐grade students. All but 2 of the participating teachers (N = 16; 10 males) identified as white. The teachers represented a range of academic subjects and varied in years of teaching experience (M = 9.80, SD = 7.93). Ninth‐grade students (N = 133) were recruited from the participating teachers' classes. Of participating students, 48.8% identified as male, 50.4% as female, and 0.8% as another gender. In addition, 63.4% of students identified as white/Caucasian, 17.1% as multiracial, 11.4% as Asian, 5.7% as Black/African‐American, 4.1% as Latinx/Hispanic, and 2.4% as another race/ethnicity.

### Quantitative Procedure

During the first week of school, participating teachers distributed parental consent forms to their ninth‐grade students (N = 417). Students whose parents indicated permission for study participation (N = 149) were then asked to provide assent to participate. Students who assented (N = 133) completed paper surveys in the auditorium at the beginning of the school year and again 5 months after baseline.

### Quantitative Measures

#### *Student‐teacher relationships*

Student‐teacher relationship quality was measured using the Classroom Student‐Teacher Relationship Scale.^52^ The 5 items (eg, “*How many of your teachers are respectful towards you?*”; α_pre_ = .84; α_post_ = .83) were measured on a 5‐point scale. Scores on each item were averaged, with higher scores reflecting higher levels of relationship quality.

#### *Motivation*

Student motivation was measured using the Academic Motivation Scale.^53^ Students responded to the stem question, “*Why do you go to high school?*,” and answers constitute 5 subscales: Amotivation (4 items; eg, “*Honestly*, *I don't know; I really feel that I am wasting my time in school*”; α_pre_ = .93; α_post_ = .83), External Regulation (4 items; eg, “*Because I need at least a high school degree in order to find a high‐paying job later on*”; α_pre_ = .91; α_post_ = .92), Introjected Regulation (4 items; eg, “*To prove to myself that I am capable of completing my high school degree*.”; α_pre_ = .91; α_post_ = .93), Identified Regulation (4 items; eg, “*Because this will help me make a better choice regarding my career orientation*.”; α_pre_ = .91; α_post_ = .85), and Intrinsic Motivation (4 items; eg, “*Because I experience pleasure and satisfaction while learning new things*.”; α_pre_ = .93; α_post_ = .95). Each item was measured on a 7‐point scale. Scores for each subscale were averaged, with higher scores reflecting higher levels of each construct.

#### *School belonging*

Student belongingness was measured using 18 items from the Psychological Sense of School Membership.^54^ Each item (eg, “*I feel like a part of my school*”) was measured on a 5‐point scale (α_pre_ = .90; α_post_ = .86). Scores on each item were averaged, with higher scores reflecting higher levels of school belonging.

#### *Student behavior*

Student behavior was measured using the Strengths and Difficulties Questionnaire.^55^ The SDQ includes 5 subscales: Emotional Symptoms (5 items; eg, “*I get a lot of headaches*, *stomach‐aches or sickness*”), Conduct Problems (5 items; eg, “*I get very angry and often lose my temper*”), Hyperactivity/Inattention (5 items; eg, “*I am restless*, *I cannot stay still for long*”), Peer Relationship Problems (5 items; eg, “*I would rather be alone than with people of my age*”), and Prosocial Behavior (5 items; eg, “*I try to be nice to other people*. *I care about their feelings*”). Each item was measured on a 3‐point scale. The Emotional Symptoms, Conduct Problems, Hyperactivity/Inattention, and Peer Relationship Problems subscales were averaged to create a Total Problems subscale (α_pre_ = .84; α_post_ = .83). Prosocial Behavior was retained as an independent subscale (α_pre_ = .66; α_post_ = .63). Higher scores reflect higher levels of each construct.

#### *Academic indicators*

Academic indicators included grade point average, earned credits, and unexcused and excused absences. Academic records for participating students were obtained from the school district at the end of the school year. Records for the year of the study (fall and spring terms) and the year prior (eighth‐grade fall term) were included in the data set. Records were not cumulative and provided independent data for each term.

### Quantitative Analytic Plan

For each program outcome, 2 repeated measures models were conducted to adjust for the nesting of time points within individuals using M*plus* 8.1.^56^ For the first model, each outcome was regressed onto a time variable (0 = *pre‐test*, 1 = *post‐test*) to examine change in student outcomes for the full sample. For the second model, a race main effect (0 = *white* students, 1 = *students of color*) was added at level 2. A cross‐level interaction was modeled to examine whether race moderated the effect of time. Twelve students did not complete the post‐test. These students reported significantly higher levels of student‐teacher relationships and school belonging at pre‐test than students who had post‐test data. Full information maximum likelihood was used to handle missing data.^57^

### Qualitative Procedure

Data for the qualitative analyses were compiled from 2 sources. First, we collected teachers' responses to the monthly PLC roster reflection, where they indicated their relationship phase—Establish, Maintain, or Restore—with each student and reflected on the distribution of race/ethnicity and gender across each phase. A total of 4 PLC meetings were held. Twelve teachers attended the first meeting, 10 attended the second, and 6 attended each of the third and fourth, for a total of 34 PLC reflections completed. Second, at the end of the school year, all teachers were invited to participate in semi‐structured interviews to provide feedback regarding the cultural responsiveness of E‐EMR. Trained research staff conducted, recorded, and transcribed phone interviews with 13 teachers, which was sufficient to reach saturation.^58^ Teachers received $50 for completing the interview.

### Qualitative Coding and Analysis

The 13 interview transcripts and 34 PLC reflections were imported into and coded with Dedoose.^59^ The goal of the coding process was to identify segments of the teacher interviews and PLC reflections where teachers explicitly reflected on how issues of bias, race/ethnicity, and culture arose in their relationship‐building practices. We also coded references to other sociodemographic factors, such as gender or language, when there was an explicit connection to our equity‐focused research questions. Four coders read a subset of 2 transcripts and 4 PLC reflections each to develop initial codes. A codebook was developed based on codes that were consistent across the sources. Two rounds of codebook development and refinement were conducted before testing for interrater reliability. Then, the first author coded 2 interview transcripts and 4 PLC reflections, and the 3 other coders assessed their reliability with the master codes. Any discrepancies between the team members' codes were discussed to come to consensus and further refine the codebook. Three rounds of testing and refinement were conducted until team members reached adequate inter‐rater reliability (κ > .72).^60^ Three coders then completed the rest of the coding, with a mid‐point meeting to address any questions (Table 2).

**Table 2:** Descriptive Statistics for Program Outcomes

		Pre‐Test						Post‐Test				
Outcomes		N	M	SD	Min	Max		N	M	SD	Min	Max
Student‐teacher relationships												
General		107	2.58	.78	.80	4.00		119	2.53	.76	.80	4.00
Relationship equity		111	2.53	.58	1.00	4.00		119	2.63	.54	1.67	4.33
Motivation												
Amotivation^1^		113	1.55	1.05	1.00	6.75		121	1.49	.72	1.00	4.00
External regulation		112	5.07	1.54	1.00	7.00		120	4.94	1.52	1.25	7.00
Introjected regulation		112	4.15	1.60	1.25	7.00		121	4.24	1.74	1.00	7.00
Identified regulation		113	5.26	1.35	1.75	7.00		119	5.19	1.26	1.50	7.00
Intrinsic motivation		112	4.78	1.54	1.00	7.00		119	4.63	1.50	1.00	7.00
School membership												
Belongingness		112	2.99	.38	2.05	3.80		119	3.23	.39	2.17	4.00
Student behavior												
Prosocial behavior		107	1.62	.34	.40	2.00		119	1.60	.33	.80	2.00
Problem behavior^1^		107	.77	.20	.32	1.30		119	.72	.19	.35	1.35
Academic indicators												
Excused absences		130	1.65	2.49	0.00	16.36		128	3.31	8.20	.00	90.00
Unexcused absences		130	1.23	1.41	0.00	6.78		128	3.73	3.15	.00	18.79
GPA		130	3.71	.50	1.17	4.00		128	3.68	.56	1.33	4.00
Earned credits		130	3.15	.32	1.25	3.25		129	3.21	.52	1.50	7.25

Note. ^1^indicates negatively valanced program items, where decreases from pre‐ to post‐ test are expected.

## RESULTS

### Quantitative Results

**Figure 1:**
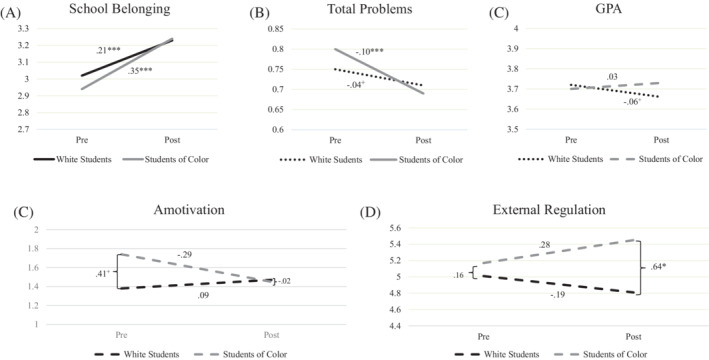
A‐E. Simple Slopes for Pre‐Post Differences for White Students and Students of Color for Significant or Marginal Race by Time Interactions. *Note*. Solid lines represent significant changes over time, dotted lines represent marginal changes over time, Dashed Lines represent non‐significant changes over time. Brackets indicate differences between white Students and Students of Color at pre‐ and post‐test. Numbers represent unstandardized regression coefficients, ***p < .001, **p < .01, *p < .05, ^+^p < .10.

Table 2 presents descriptive statistics for program outcomes at pre‐ and post‐test. Two models were conducted for each outcome to determine whether students scored differently on program outcomes from pre‐ to post‐test, and whether these differences were moderated by race (Table 3). In the time‐only model, students reported significant (p < .05) increases in student belongingness, marginal (p < .10) increases in prosocial behavior, and significant decreases in problem behavior over time. The number of excused and unexcused absences increased significantly from the fall to spring semester. When race and the race by time interaction were added to the model, time no longer marginally predicted prosocial behavior, but did marginally predict an increase in GPA. Race main effects indicated that students of color demonstrated higher levels of amotivation and earned credits, but lower levels of unexcused absences, excused absences, and prosocial behavior, compared to white students. A marginal or significant race by time interaction was identified for 5 outcomes—belongingness, total problems, GPA, amotivation, and external regulation—with changes from pre‐ to post‐test favoring students of color (Figure 1).

**Table 3:** Results of 2‐Level General Linear Models to Examine for Pre‐ to Post‐test Differences for Participants (N = 133)

	Model 1						Model 2											
	Time (pre = 0)			Grade 8			Time Main Effect (Pre = 0)			Race Main Effect (White = 0)			Race*Time Interaction			Grade 8 Scores		
Outcomes	β	SE	p	β	SE	p	β	SE	p	β	SE	p	β	SE	p	β	SE	p
Student‐teacher relationships																		
Positive relationships	−0.06	.07	.37	—	—	—	−0.02	.08	.82	−0.23	.16	.17	0.08	.14	.56	—	—	—
Motivation																		
Amotivation^1^	−0.09	.11	.40	—	—	—	0.08	.09	.34	**0.45**	**.25**	**.07**	**−0.47**	**.25**	**.05**	—	—	—
External reg.	−0.02	.15	.91	—	—	—	−0.20	.19	.30	0.16	.28	.58	**0.52**	**.29**	**.08**	—	—	—
Introjected reg.	0.20	.17	.23	—	—	—	0.22	.20	.26	0.42	.31	.16	−0.02	.33	.96	—	—	—
Identified reg.	0.01	.13	.96	—	—	—	−0.15	.17	.38	0.30	.23	.21	0.39	.24	.10	—	—	—
Intrinsic mot.	−0.03	.14	.81	—	—	—	−0.17	.16	.27	−0.01	.28	.99	0.40	.28	.16	—	—	—
School membership																		
Belongingness	**0.27**	**.04**	**<.001**	—	—	—	**0.23**	**.04**	**<.001**	−0.11	.10	.27	**0.13**	**.06**	**.02**	—	—	—
Student behavior																		
Prosocial beh.	**0.07**	**.04**	**.07**	—	—	—	0.01	.04	.78	**−0.20**	**.08**	**.01**	0.06	.07	.41	—	—	—
Problem beh.^1^	**−0.07**	**.02**	**<.001**	—	—	—	**−0.05**	**.02**	**.01**	0.04	.05	.38	**−0.06**	**.04**	**.09**	—	—	—
Academic indicators																		
Excused absences	**1.02**	**.22**	**<.001**	**.35**	**.04**	**<.001**	**0.99**	**.32**	**.002**	**−0.73**	**.28**	**.01**	0.12	.39	.77	**.30**	**.05**	**<.001**
Unexcused absences	** 2.43**	**.28**	**<.001**	**.45**	**.10**	**<.001**	**2.75**	**.39**	**<.001**	−0.19	.29	.51	−0.37	.49	.45	**.42**	**.12**	**<.001**
GPA	−0.03	.02	.22	**.80**	**.07**	**<.001**	**−0.06**	**.03**	**.07**	0.03	.05	.57	**0.09**	**.04**	**.04**	**.78**	**.08**	**<.001**
Earned credits	0.05	.05	.32	.15	.71	.83	0.04	.05	.48	**0.13**	**.06**	**.03**	0.04	.11	.71	.07	.10	.51

Note. ^1^indicates negatively valanced program outcomes, where decreases from pre‐ to post‐ test are expected.

Reg = regulation, Mot = motivation, Beh = behavior.

Bolded font represent significant or marginal (p < .10) pre‐post differences.

### Qualitative Results

The qualitative coding process resulted in the development of 2 distinct types of codes: content and social identity (Table 4). Content codes captured the topic or theme a teacher discussed, whereas social identity codes captured the socio‐demographic characteristics a teacher referred to, such as race/ethnicity, gender, or language. Social identity codes were developed to provide context regarding teachers' reflections on the content code themes, so these codes are subsumed within our discussion of the content codes below.

**Table 4:** Qualitative Codebook

		Frequency		
	Definition and Quotes	Overall 122 total excerpts	Female N = 6 (40%)	White N = 13 (87%)
Content codes				
E‐EMR strategies and structures	Apply this code when a teacher is discussing Explicit Establish‐Maintain‐Restore (EMR) strategies or structures (eg, professional learning communities [PLCs], reflections, having a common language for relationship building). For strategies, code when a strategy is being discussed, regardless of whether it is explicitly named. *“Yeah*, *talking about regular human stuff*, *and just spending time*. *Humor*, *in the Banking Time one*. *Making sure that you learn and appreciate individual students' sense of humor if they're willing to share that with you*, *goes an awful long way*. *It's necessary for me too*. *Not just for the student*. *Yeah*, *Banking Time I think pretty much up front*, *I think particularly with students of color that are really suspicious of white male authority figure*, *and that often presents fairly earlier in the year*. *Figuring out who the students that you're going to need to learn at a more personal level*, *is pretty obvious for some*, *and it works pretty well*.*”* “*I would note within Ownership of the Problem is also acknowledging or saying like*, *I realize I may have been influenced by implicit bias and*, *or I did something ignorant as a white person and I*… *owning race and identity is a part of that*, *Ownership of the Problem is particularly important*.*”*	60 (49%)^*^	22 (37%)^†^	47 (78%)^‡^
General care for students	Apply this code when a teacher is discussing their intention (does not need to include intent to specific action), motivation, and general practice for caring for, listening to, and getting to know students. These are not references to specific strategies, but to the importance of building relationships and getting to know and valuing students' perspectives, letting students know they're seen. “*My class is very white*. *My 2 African American students I worked intentionally to get to know very early on ‐ they've been M all along*. *My African student same thing*. *My 4 Asian students are M* (3) *or R* (1). *Again*, *I was intentional from day 1 to build a rapport with them*.*”*	18 (15%)	7 (61%)	14 (78%)
Teacher identity	Apply this code when a teacher is discussing their identity or role as a teacher. This can include their own biases, identity, or cultural background. This can also include how they view themselves as a professional in their role as a teacher, and how this impacts the way they interact and relate to their students. Also, apply this code if teachers discuss interactions/dynamics amongst teachers. *“But with the Empathy Statements I really need to think about my positionality and not try to come off as either privileged or condescending*. *It's pretty easy*, *I'm a white male*, *for me to do that*. *So*, *if I sit down and I say*, *“I'm really sorry that's happening*,*" if I don't get the tone right*, *it sounds really off*. *And I try to be sensitive too to the fact that I don't know a lot about* … *well*, *there's a lot about the lived experience of being a student of color that I just don't know*. *And I tend to empathize with*. *And so*, *yeah*, *that's tricky*. *Worth doing though*.*” In response to the PLC equity reflection*, *another teacher reflected*, *“Although this class is majority black*, *it still appears those are the students that I need to establish and restore the most*. *Being a white teacher*, *I may feel like less of an ally to them*.*”*	40 (33%)	10 (25%)	23 (58%)
Classroom/school instruction and climate	Apply this code if teachers refer to ways that they use classroom practices (eg, instructional practices, curricular activities, classroom management strategies) to address equity, race, culture, or bias. Also use this code if teachers discuss the climate (eg, student–student interactions, tensions) or composition of their classroom or school with explicit regard to equity, race, culture, or bias. “*A lot of it is intentionally how I structure lessons*, *how I structure teaching*, *how we introduce an idea always so that no matter what somebody's math background is*, *the way that a new topic or idea is introduced is something that's accessible to every student in the class so that everybody feels like they have an opportunity to be successful*. *Because that's what's really needed*, *especially with students of color who haven't been successful and feel very labeled as an unsuccessful student or like a disruptive student*. *Just making sure that they can see that*.*”*	35 (29%)	18 (51%)	28 (80%)
Power, privilege, systems of oppression	Apply this code when a teacher explicitly refers to systems of power, privilege, and/or systemic racism, and how these dynamics impact student‐teacher relationships. Include use of oppressive terms and phrases and movements, individual and systemic level racism, and societal references to historical system of oppression that could be beyond the education system. *“I think the combination of letting go of the previous interaction and the ownership of the problem worked well with that student because we had an incident during Black Lives Matter week*, *it's a white student that he made a comment that I didn't think was appropriate and he was really upset with me and we had a whole discussion about it after*, *which I think left a really bad taste in my mouth*. *But the next day*, *we just let go of the previous interaction*. *I told them that I could have verbalized how I*, *why I thought his comment was inappropriate better*, *and we kind of moved on*. *So I feel that incident could have really tanked our whole relationship*. *But it didn't*.*”*	16 (13%)	4 (25%)	11 (69%)
No changes/pattern	Apply this code if a teacher indicates that they made no changes to their relationship‐ building practices for students of different ethnic/racial groups or if they did not notice or show evidence of any differential patterns in relationship quality or strategies according to race/ethnicity.	21 (17%)	10 (48%)	18 (86%)
Social identity codes	Apply any of these codes in addition to (or regardless of) the content codes above to indicate if the teacher is discussing a particular social group/identity when referring to students, teachers, themselves, or classroom context. Never code Social Identity in isolation from the sub‐codes.	108 (89%)	44 (41%)	82 (76%)
Race	Apply this code if teachers refer to a general racial/ethnic group (eg, students of color)	70 (57%)	27 (39%)	50 (71%)
Specific race**/**ethnicity	Apply this code if teachers refer to a specific racial/ethnic group (eg, Asian students, White boys), as opposed to referring to race/ethnicity generally, or using broad phrases like “students of color”	39 (32%)	18 (46%)	28 (72%)
Gender/sex	Apply this code if teachers are referring to gender/sex of students or themselves	52 (43%)	23 (44%)	42 (81%)
Language	Apply this code if teachers reference students' home, native, or first language (eg, English Language Learners)	10 (8%)	5 (50%)	9 (90%)
Sexual orientation	Apply this code if teachers reference to sexual orientation of students or themselves	4 (3%)	0 (0%)	2 (50%)
Social economic status	Apply this code if teachers reference students' social economic status	1 (0.8%)	0 (0%)	1 (100%)
Ability	Apply this code if teachers reference students' disability status	4 (3%)	2 (50%)	3 (75%)
Religion	Apply this code if teachers reference students' religion	3 (2%)	1 (33%)	3 (100%)

^*^ In this column, percentages represent the percentage of excerpts (out of 122) to which the code was applied.

^†^ In this column, percentages represent the proportion of excerpts receiving the code overall that were associated with interviews/reflections from female teachers. For example, of the 60 EMR Strategies and Structures codes, 22, or 37%, were from interviews/PLC reflections of female teachers.

^‡^ In this column, percentages represent the proportion of excerpts receiving the code overall that were associated with interviews/reflections from white teachers. For example, of the 60 EMR Strategies & Structures codes, 47, or 78% of them were from interviews/PLC reflections of white teachers.

#### *E‐EMR strategies and structures*

Teachers provided reflections regarding how the E‐EMR strategies and structures addressed issues of equity and bias in their relationships with students. When asked which strategies helped facilitate strong relationships with students of color, many teachers referred to the *Banking Time* Establish strategy and the Restore strategies, including *Collaborative Problem Solving* and *Ownership of the Problem*. Teachers also discussed how relationship status across the Establish, Maintain, and Restore phases differed for various student groups. Some teachers noted it was more difficult to establish relationships with students who did not share similar identities/backgrounds as themselves. Other teachers focused most on establishing relationships with students from marginalized backgrounds who they perceived to be most vulnerable, so they had the weakest relationships with students from more privileged backgrounds.

#### *General care for students*

Teachers also sometimes discussed equity in student‐teacher relationships more broadly, without referencing E‐EMR specifically. This included teachers reflecting on the strong relationships they built with particular student groups, without indicating which practices they engaged in to do so. Similarly, teachers expressed their intentions to build stronger relationships with certain student populations but did not suggest a practice they might use to reach this goal. Finally, teachers discussed practices they used to integrate a focus on equity into their student‐teacher relationships, but these practices were not specific to E‐EMR. It is important to note that this code was applied much less frequently than the *E‐EMR Strategies & Structures* code (18 compared to 60), suggesting teachers were inclined to discuss equity in relationship building within the context of the E‐EMR framework.

#### *Teacher identity*

Teachers reflected on how their own background and identity, typically their race and gender, impacted their relationships with students. They also discussed how their role as a teacher, including the power dynamics inherent within the student‐teacher relationship affected their interactions with students. It is important to note that although white teachers comprised 87% of the study sample, only 56% of the *Teacher Identity* codes were applied to excerpts from white teachers' PLCs and interviews. This is the largest discrepancy between participant demographics and application of any of the content codes. Although both white teachers and teachers of color discussed how their identities impacted their relational practices with their students, teachers of color also discussed their identity in relation to other staff and how that intersection affected both their dynamics with their students and other staff.

#### *Classroom/school instruction and climate*

Teachers also noted how individual relationships were impacted by the larger classroom or school context, or how their relationships were connected to other processes within their classes. This suggests that teachers did not perceive individual student‐teacher relationships to be isolated from other aspects of their teaching practices/responsibilities or from the context of other students. Some teachers discussed how they were able to integrate relationship building into their instructional practices. By learning more about their students' cultural backgrounds (eg, home language) or racialized experiences (eg, stereotypes regarding ability) using Establish strategies, they felt better able to address their students' academic needs. Teachers also described how racial/gender composition or dynamics of certain classes influenced how they built relationships with particular students.

#### *Systems of power, privilege, and oppression*

At times, teachers discussed how their relationships with students were situated within larger societal systems of power and privilege. This included references to social processes (eg, gentrification) or movements (eg, Black Lives Matter) that teachers addressed with students, which served as the backdrop for relationship building. In addition, teachers reflected on how incidents of racism, sexism, homophobia, and anti‐Semitism that arose amongst the student body impacted individual relationships. Finally, teachers described how their relationship‐building practices were influenced by systems of power, including perceptions of their or students' amount of privilege. This included feeling challenged building relationships with students whose teachers thought had high levels of privilege and/or wanting to provide supports for students who faced systemic disparities and discrimination that impacted their educational trajectories. This also included teachers changing their behavior or practices based on feedback from students regarding systems of power and privilege (eg, asking students about their preferred pronouns).

#### *No changes/patterns*

The above themes capture the ways teachers discussed integrating cultural responsiveness and racial equity lenses into their relationship‐building practices. In contrast, it is important to note that sometimes (17% of excerpts), teachers indicated that their relationship‐building practices and relationship status did not differ with students according to race/ethnicity or other socio‐demographic factors, even when directly probed.

## DISCUSSION

Student‐teacher relationships play an important role in supporting students' social‐emotional and academic wellbeing and enhancing the social and emotional climate of schools, a core component of the WSCC framework. The current study conducted a mixed‐methods feasibility evaluation of a promising equity‐explicit student‐teacher intervention (E‐EMR) for ninth‐grade students. Given disparities in student‐teacher relationship quality and educational outcomes for students of color, we intentionally integrated an equity lens into intervention content and the mixed‐method evaluation of this study. Thus, this study emphasizes the importance of an explicit focus on racial equity when applying different domains of the WSCC framework.

### Changes in Student Outcomes

Quantitative analyses demonstrated that E‐EMR may have a buffering effect for students of color. Specifically, relative to white students, students of color showed greater improvement from pre‐ to post‐test on school belongingness, total problems, amotivation, external regulation, and GPA. Previous research suggests that student‐teacher relationship quality, effort, and work completion are likely to decline over the course of the academic year.^61^ Although these results are preliminary, this suggests the promise of E‐EMR for narrowing longstanding gaps in academic and social‐emotional outcomes across racial and ethnic lines. A recent systematic review^62^ showed that only 19% of educational intervention studies tested the effectiveness of the intervention for reducing racial/ethnic disparities, and less than half of these displayed promise of reducing such gaps. More work is needed to develop interventions that can reduce disparities across multiple domains of school health, including mental, physical, and psychological well‐being; it is likely that incorporating equity‐explicit intervention content, such as strategies for reducing implicit bias, is important to ensure that interventions are effective for those who may need them most.^45^ Currently, the WSCC framework does not include an explicit focus on equity to ensure that interventions selected are designed and deployed in ways to disrupt longstanding disparities and promote more equitable outcomes for all students.

Although it is difficult to interpret the main effects of time due to the lack of a control group, we found a deterioration from pre‐ to post‐test on some academic indicators (GPA, absences) and motivation, particularly for white students. Unfortunately, this decline is consistent with prior research showing decreases in student engagement, motivation, and academic performance after the ninth‐grade transition.^63^, ^64^ In the context of this normative downward trend, interventions such as E‐EMR may reduce deterioration and protect against certain risks, which may preserve students' academic engagement and performance during critical developmental transitions. Future research that includes a control group can identify whether E‐EMR in fact does buffer against even more severe declines.

It is important to note that we did not find significant changes on our measure of student‐teacher relationship quality. This may be because our measure of student‐teacher relationships was quite broad, asking about students' relationships with all of their teachers. We only trained teachers whose teaching load was greater than 50% ninth‐graders, and most students in this study had other teachers who did not meet this criterion. We opted for this measure because more widely‐used measures of student‐teacher relationships^65^ have not been validated with high school students. Measuring student‐teacher relationships in high school is more challenging than in younger grades, as students interact with more teachers and switch classes more often. Future research should include measures of relationship quality that ask students about specific teachers, especially those who are intentionally implementing student‐teacher relationship interventions.

### Teachers Perceptions of Equity within Relationship Building

Qualitative analysis revealed that teachers often incorporated issues of equity into their relationships with students. Teachers focused on learning about students' cultures, home lives, and interests, which enhanced their ability to build relationships and resolve conflicts. Some teachers noted that by learning about students' backgrounds, they were also better able to genuinely engage students in academic work. This is consistent with previous research that demonstrated how subject matter learning can be enhanced through culturally responsive practices that reduce inconsistencies between home and school contexts.^66^, ^67^ Some teachers even reported that because their focus was to build positive relationships with students from historically marginalized groups, their relationships with students from privileged groups remained in the Establish phase. This may be due to the high number of students a high school teacher has and the limited time they can devote to implementing relationship‐building practices with students.^68^ In contrast, some teachers did note that it was more difficult to build relationships with students with identities/backgrounds different from their own. Extant research has suggested that feelings of closeness and trust are more difficult to foster in cases of racial/ethnic and cultural misalignment between students and teachers,^32^, ^69^, ^70^ though this has more often been explored from the student's, as opposed to the teacher's, perspective. Notably, multiple teachers commented on the importance of owning their own racial privilege, implicit bias, or position of power as a teacher. These reflections indicate teachers were able to explicitly focus on identity characteristics (students' and their own) as they built and maintained relationships with students.

Some insights gleaned during qualitative analysis may serve as targets for future intervention development. First, in 17% of excerpts, even when directly probed, teachers stated that their relationship practices did not differ according to socio‐demographic factors. This may indicate a need for even greater emphasis in the E‐EMR training and PLCs on the importance of incorporating responsiveness to ethnicity/race, gender, and other identity characteristics in relationships with students. Second, although white teachers comprised 87% of the study sample, only 56% of the *teacher identity* codes were applied to excerpts from white teachers. E‐EMR could highlight through supportive discussion the reality that white teachers are more likely to overlook their own identities and how their positionality in their role and identity influences relationships with students.^71^, ^72^, ^73^, ^74^ Relatedly, teachers of color discussed their identities in relation to other teachers during PLCs, including feelings of exhaustion/frustration being in the position of “teaching white teachers about how to deal with students of color.” E‐EMR and other relationship programs can help acknowledge these dynamics and offer practical suggestions for how to avoid placing the brunt of this responsibility on teachers of color.^75^, ^76^ Employee wellness is a core component of the WSCC framework; it is crucial to understand how intrinsic and interpersonal processes amongst teachers may promote or impair educator well‐being, with an eye toward reducing systemic inequities faced by teachers of color.^77^ Although the primary focus of E‐EMR is on improving student outcomes, the intervention also has the potential for improving teacher wellness, especially with additional, intentional adaptations to the training and implementation supports.

### Integrated Conclusions and Future Directions

Equity‐Explicit Establish‐Maintain‐Restore demonstrates promise for increasing racial equity, evidenced by larger pre‐post gains in student outcomes for students of color and by teachers' reflections on how they incorporated a focus on equity into their relationship‐building practices. Unfortunately, due to the limited teacher sample size and the structure of data collection, we were not able to examine the direct relations and interactions between teacher and student outcomes; however, it is promising to see some evidence of potential mechanisms of change (ie, teacher behavior) that likely contribute to improvements demonstrated in student outcomes.

In addition, there are some notable patterns that emerge across both quantitative and qualitative sources. In particular, both the student and teacher data speak to the embeddedness of the individual relationship within broader contexts. This was exemplified by significant changes from pre‐ to post‐test in students' ratings of more global constructs (ie, school belonging, academic motivation), and by teachers' reflections on how individual relationships were situated within the context of their own identity, their classroom/school environment, and larger societal systems. Although the E‐EMR strategies target individual interactions, the intervention may affect students' and teachers' conceptualizations of the contexts in which they are embedded. This is consistent with the nature of the intervention, which is delivered to a grade‐level team of teachers and includes a collaborative PLC component. This is also consistent with previous research which has emphasized how organizational factors within the school and broader social dynamics influence and are intertwined with student‐teacher relationships, especially at the high school level.^35^, ^78^, ^79^, ^80^ Ensuing research should continue to explore the implications of student‐teacher relationship practices and interventions for enhancing not only individual interactions, but school health more broadly. For example, interventions such as E‐EMR can target interactions beyond the classroom; relationship‐building and equity strategies can be enacted by school nurses, counselors/psychologists, coaches, and other school staff, as well as in collaboration with communities and families, to facilitate well‐being across multiple domains of functioning and in multiple educational contexts. Applications of interventions in this way would be consistent with the WSCC framework by extending efforts to promote student wellbeing across stakeholders within schools and outside of schools into the community. Future studies can also explore the importance of the intersection between student and teacher identity from both the student and teacher perspective. Teachers often discussed how alignment or misalignment between their own race/gender and that of their students may influence relationships, but this was not quantitatively addressable with the current data.

### Limitations

Although this study provides important insights regarding the effects of an equity‐explicit student‐teacher relationship intervention, it is not without limitations. The primary limitations lie in the study design. First, this was a preliminary study, using a pre‐post design without a control group. Therefore, we are unable to understand whether the changes we observed are due to the intervention or unrelated change over time. In addition, our student recruitment approach did not intentionally sample for students according to race/ethnicity, so we were unable to conduct disaggregated analyses for particular subgroups. Furthermore, we were not able to sample students in a manner that would allow us to align student ratings with particular teachers; this would have provided more information regarding the connections between specific teacher perspectives and the changes observed for their students. Finally, our limited teacher sample size prohibited any quantitative analyses for teachers. Despite these limitations, this study demonstrates potential promise of the E‐EMR approach, and these methodological concerns can be addressed in a more robust evaluation. In addition, the rigorous mixed‐methods design employed in this study can buoy some methodological limitations, by triangulating findings from multiple stakeholder groups and analysis approaches. Mixed‐methods approaches are especially important during pilot studies, as they can provide insight for future more rigorous and well‐powered evaluations.^81^, ^82^, ^83^

## IMPLICATIONS FOR SCHOOL HEALTH

Consistent with the WSCC framework, a core feature of health and wellbeing in school is social and emotional climate. Climate reflects how students perceive their experiences in school,^84^ and one major source of students' experience is their interactions with teachers. Intentionally selecting and implementing programs that allow teachers to learn about, reflect on, and plan for how to build, maintain, and restore positive relationships with students offers a promising approach to improving school climate and promoting more equitable student outcomes. From an implementation perspective, it is critical for school leaders to allocate resources, protected time, and professional development opportunities to student‐teacher relationship interventions to support educators to adopt and deliver such interventions with fidelity and ensure that students can benefit from them.^85^ Coordinated and collaborative efforts, with sufficient resources, training, and accountability have shown to be essential for successfully implementing WSCC initiatives within schools and districts.^86^

This study represents the ongoing efforts among school researchers and practitioners to ensure interventions are designed and delivered in ways to address longstanding disparities for students of color. Without an intentional focus on racial equity, there is a risk that well‐intended programs/practices do not address the backgrounds of students of color^37^ and continue to advantage more privileged students who already have adequate health, wellbeing, and performance in school.^87^, ^88^ Highlighting the perspectives of diverse stakeholders when developing and testing interventions can ensure they are designed and refined in developmentally and culturally responsive ways.^49^ These methods increase the likelihood that schools can identify and implement interventions across various WSCC domains that not only work for all students, but also reduce pervasive biases and disparities that persist and continue to undermine educational outcomes for students of color.

### Human Subjects Approval Statement

This study was approved by the University of Washington Institutional Review Board (STUDY00002720).

### Conflict of Interest

Dr. Cook receives consultative payment by school districts to support the adoption and implementation of EMR. He receives roughly $3000 in stipends for delivering the training and providing follow‐up technical assistance to teachers, administrators, and professional support staff.
